# Improving Generalization in Collision Avoidance for Multiple Unmanned Aerial Vehicles via Causal Representation Learning †

**DOI:** 10.3390/s25113303

**Published:** 2025-05-24

**Authors:** Che Lin, Gaofei Han, Qingling Wu, Boxi Wang, Jiafan Zhuang, Wenji Li, Zhifeng Hao, Zhun Fan

**Affiliations:** 1 Department of Electronic Engineering, Shantou University, Shantou 515063, China; 19clin1@stu.edu.cn (C.L.); 22gfhan@stu.edu.cn (G.H.); 22qlwu@stu.edu.cn (Q.W.); 19bxwang@stu.edu.cn (B.W.); liwj@stu.edu.cn (W.L.); haozhifeng@stu.edu.cn (Z.H.); 2 Shenzhen Institute for Advanced Study, University of Electronic Science and Technology of China, Shenzhen 518000, China

**Keywords:** deep reinforcement learning, multi-UAV collision avoidance, generalization failure, causal representation learning, causal intervention

## Abstract

Deep-reinforcement-learning-based multi-UAV collision avoidance and navigation methods have made significant progress. However, the fundamental challenge of those methods is their restricted capability to generalize beyond the specific scenario in which they are trained on. We find that the cause of the generalization failures is attributed to spurious correlation. To solve this generalization problem, we propose a causal representation learning method to identify the causal representations from images. Specifically, our method can neglect factors of variation that are irrelevant to the deep reinforcement learning task through causal intervention. Subsequently, the causal representations are fed into the policy network for action prediction. Extensive testing reveals that our proposed method exhibits better generalization results compared to state-of-the-art methods in different testing scenes.

## 1. Introduction

The low-altitude economy, as a strategic emerging industry with broad prospects, is experiencing rapid development, with the application of unmanned aerial vehicles (UAVs) becoming increasingly widespread. In fields such as environmental monitoring [1], emergency rescue [2,3], intelligent logistics [4], and precision agriculture [5], UAVs have already demonstrated significant social and economic value. Collision avoidance and navigation capabilities are crucial in ensuring that UAVs operate safely and effectively. UAVs need to navigate around obstacles in intricate environments and find the best route from a starting point to a destination point.

Unmanned aerial vehicle collision avoidance and navigation have been widely studied. Conventional methods typically utilize simultaneous localization and mapping (SLAM) to build a local representation of the environment, which is then used for planning safe paths [6,7,8]. Another approach is path retracing [9], where UAVs follow routes that are either predefined or learned from demonstrations. Despite their success in structured environments, these techniques often struggle to adapt to dynamic or unfamiliar scenarios. A key limitation is their reliance on pre-built maps and handcrafted features, which restricts their robustness, flexibility, and generalization in real-world applications.

To address the limitations of traditional methods, deep reinforcement learning (DRL) has emerged as a promising alternative [10,11,12]. DRL enables UAVs to autonomously learn effective navigation strategies in complex and dynamic environments, eliminating the need for manual rule design or parameter tuning. It works by extracting compact state representations from raw sensory inputs, selecting actions based on these states, and refining its policy or value estimates through feedback in the form of rewards or penalties from the environment. The ultimate goal is to learn an optimal strategy that maximizes cumulative long-term rewards and achieves the desired task performance.

**Figure 1:**
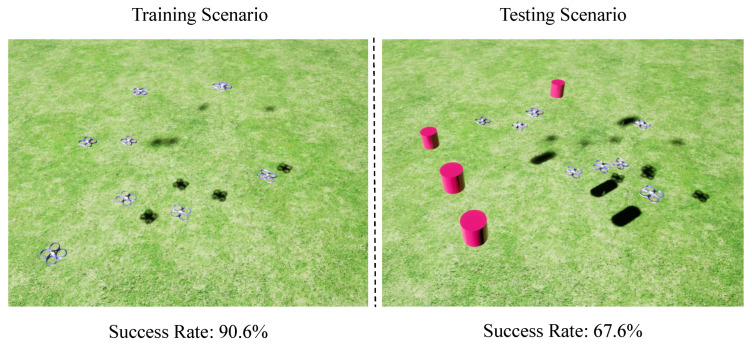
Illustration on the influence of spurious correlations. Due to the presence of spurious correlations, deep reinforcement learning algorithms often over fit to the specific obstacles encountered during training. As a result, these algorithms may achieve high success rates in the training scenario but develop a fragile policy that struggles to adapt to unseen obstacles in testing scenarios.

Despite the potential of deep reinforcement learning (DRL), a key limitation lies in its poor generalization to scenarios beyond the training environment. As illustrated in Figure 1, consider a case where UAVs are trained in a simplified environment containing only UAV-like obstacles. In this setting, they learn to navigate from a starting point to a target location by avoiding these familiar obstacles. However, when deployed in a test environment with novel, previously unseen obstacles, the learned policy—while effective in the original training domain—shows a significant drop in performance. This example highlights a major challenge in current DRL-based navigation systems: their limited ability to generalize to environments that differ from the training distribution.

To reveal the reasons for the generalization failures in reinforcement learning, we review pioneering work in the field of multi-UAV collision avoidance based on deep reinforcement learning (i.e., SAC + RAE [13]). We have discovered that spurious correlations are the root of the generalization failures. Specifically, the DRL algorithms often overfit to the specific obstacles encountered during training. As a result, these algorithms may achieve a high success rate in the training scenario but develop a fragile policy that struggles to adapt to unseen obstacles in testing scenarios. Furthermore, learned policies often struggle to ignore non-causal factors (e.g., the obstacle shape) in their sensor observations and exhibit strong sensitivity to variations in these factors.

Recent studies also show that the generalization of DRL policies can be improved substantially by causal representation learning [14,15]. Causal representation learning [16] aims to find the high level causal variables from low-level observations. It can effectively extract causal factors that impact the task, thereby reduce the effect of spurious correlations on the generalization performance of DRL. Therefore, we propose a causal representation learning (CRL) approach to identify the causal representations from images. Specifically, we only intervene the shape of obstacles within deep images, keeping the rest content of the image unchanged. Then, the images of spherical obstacles and cubic obstacles are used solely as auxiliary task for causal representation learning. By utilizing its auxiliary task, CRL enhances its generalization ability by maximizing the mutual information across the latent representations of different obstacle images. In addition, we apply supervision signals to the latent representations to reduce redundancy between dimensions. Intuitively, this encourages different dimensions to capture distinct information. After that, CRL can effectively identify and discard irrelevant variation factors, significantly enhancing the model’s generalization capability.

To evaluate the generalization ability of CRL, we conduct several testing scenarios across different obstacles. In comparison with previous state-of-the-art (SOTA) methods, the results demonstrate that CRL outperforms the SOTA across different testing scenarios and demonstrate the efficacy of our CRL method. It is worth noting that an extended version of this paper was presented at the 2024 10th International Conference on Big Data and Information Analytics (BigDIA), Chiang Mai, Thailand, 25–28 October 2024 [17].

## 2. Related Work

### 2.1. Drl-Based UAV Collision Avoidance and Navigation

Deep reinforcement learning (DRL) offers a powerful framework for enabling autonomous obstacle avoidance and navigation in unmanned aerial vehicles (UAVs), particularly within complex and dynamic environments [18,19]. Foundational approaches often utilize Deep Q-Networks (DQNs) [20]; however, standard DQNs can encounter challenges related to training stability and convergence. To address these limitations, enhanced variants such as Double DQN [21,22] and Dueling DQN have been developed, demonstrating improved performance. Alternatively, policy gradient methods, notably Proximal Policy Optimization (PPO) [23,24], are frequently favored due to their relative stability and sample efficiency, making them robust choices for training reliable policies. For tasks involving the continuous control spaces typical of UAV flight, actor–critic architectures are particularly suitable. Algorithms like Deep Deterministic Policy Gradient (DDPG) [25] and Soft Actor–Critic (SAC) [13] effectively manage continuous actions by balancing policy exploitation with sufficient exploration. Furthermore, overcoming challenges in training complexity and bridging the simulation-to-reality gap often involves employing techniques such as curriculum learning, transfer learning, and sim-to-real strategies like domain randomization. Recognizing that real-world UAV operations often involve incomplete state information, we formulate the obstacle avoidance navigation task as a partially observable Markov decision process (POMDP). However, owing to the limitations in feature representation, the above methods are unable to generalize beyond the specific scenario in which they are trained on. Therefore, the collision avoidance navigation methods based on DRL face substantial challenges before they can be widely adopted and applied in practice.

### 2.2. Causal Representation Learning

Our work builds on the nascent field of causal representation learning [16], which is a method of modeling the causal relationships within data. It can reduce the effect of spurious correlations and transform the data into a structured representation that aligns with physical laws. Yang et al. [26] propose a method called CausalVAE, which is the first to introduce structural causal models into representation learning. By learning the causal structure of the data, CausalVAE can generate counterfactual data that differs from the training data, which helps improve the model’s generalization ability. However, CausalVAE introduces additional causal layers and structures, which increases the model’s complexity and may make the training and optimization process more challenging. To address the problem of learning causal representation from multiple distributions, Zhang et al. [27] propose applying sparsity constraints to the latent variable graph structure. By learning the causal relationships of latent variables, it can reveal the underlying causal structure of the data to understand system behavior and make decisions; while the method can learn causal relationships, interpreting these relationships requires domain knowledge, especially when dealing with complex systems and multiple latent variables. To address the sparsity of supervisory signals and the long-tail problem in causal representation learning, Zhao et al. [28] propose a general framework called DCCL, which learns disentangled causal embeddings through contrastive learning based on causal mechanisms and causal graphs. This approach can notably improve the precision and robustness of model predictions. Although DCCL enhances the handling of sparse data through contrastive learning, its performance still relies to some extent on the quality and diversity of the training data.

In this work, we conduct causal intervention on the shape of obstacles to extract invariant causal representations inspired by the above studies. This method can effectively reduce the effect of spurious correlations on the generalization ability of DRL.

## 3. Approach

### 3.1. Definitions of Some Key Notations

In order to present our method more clearly, we define some key notations in this subsection. The definition results are shown in Table 1.

### 3.2. Problem Formulation

#### 3.2.1. Survey of Deep Reinforcement Learning in UAV Collision Avoidance

Deep reinforcement learning (DRL) provides an advanced methodological foundation for facilitating collision-free navigation of unmanned aerial vehicles (UAVs) in complex environments. In this scenario, the UAV engages with the environment by executing actions informed by its real-time observations and subsequently receives feedback in the form of rewards or penalties, reflective of its operational efficacy. The primary goal is to maximize the cumulative reward over time, which drives the UAV to develop an optimal policy for navigation and collision avoidance.

Mathematically, the UAV collision avoidance problem is formalized as a partially observable Markov decision process (POMDP), which is defined by a 6-tuple (S,A,P,R,Ω,O), where:

- S denotes the state space, representing all possible configurations of the UAV and its environment.
- A represents the action space, consisting of all feasible actions that the UAV can execute.
- P is the transition probability function, $P(s^{\prime }|s,a)$, which specifies the probability of transitioning to state $s^{\prime }$ given the current state *s* and action *a*.
- R is the reward function, R(s,a), which assigns a scalar reward for taking action *a* in state *s*.
- Ω is the observation space, comprising all possible observations that the UAV can make.
- O is the observation function, O(o|s), which provides the probability of observing *o* given the true state *s*.

The UAV’s objective is to learn a policy π(a|o) that maps observations to actions, with the goal of maximizing the expected cumulative reward.

#### 3.2.2. Observation Space

The observation space O of the UAV is defined as O=[I,V,G], where:

- *I* represents the accumulation of four consecutive depth images captured by the UAV’s onboard camera. These images provide spatial information about the environment and obstacles.
- *V* denotes the current velocity of the UAV, which includes its forward, turning, and climbing velocities.
- *G* represents the Euclidean distance from the UAV’s current position to the target destination. This information helps the UAV navigate towards the goal.

#### 3.2.3. Action Space

To ensure smoother and more controllable UAV movement, the action space is defined as a set of constrained velocities in continuous space. The action vector $a=[v_{x}^{\mathrm{cmd}},v_{z}^{\mathrm{cmd}},v_{\omega }^{\mathrm{cmd}}]$, generated by the policy network π(s), consists of two linear velocities and one angular velocity. Specifically, we constrain the forward velocity to $v_{x}^{\mathrm{cmd}}\in \left(0.0,2.0\right)$ m/s, the lateral velocity to $v_{z}^{\mathrm{cmd}}\in \left(-0.5,0.5\right)$ m/s, and the angular velocity to $v_{\omega }^{\mathrm{cmd}}\in \left(-0.5,0.5\right)$ rad/s. Notably, backward motion ($v_{x}^{\mathrm{cmd}}<0$ m/s) is disallowed, as each UAV is equipped with a front-facing camera only, which lacks rear visibility and cannot assist in avoiding collisions during backward movement.

#### 3.2.4. Reward Function

The purpose of collision avoidance navigation is to control the UAVs to reach the target position in a complex environment without collision. This process can be divided into two subtasks: target approach $r_{g}$ and obstacle avoidance $r_{c}$. Therefore, when the UAVs safely reaches the target positions, it should be given positive feedback as a reward. Conversely, when a collision occurs, negative feedback should be given as a punishment.

(1) $$r=r_{g}+r_{c}$$

(2) $$r_{g}=\left\{\begin{array}{c} r_{arrival}\;\;\;\;\;\;\;\;\;if\;d_{t}<0.5\\ \alpha_{goal}\cdot \left(d_{t-1}-d_{t}\right)\;\;\;\;\;otherwise \end{array}\right.$$

where $d_{t}$ is the euclidean distance at time t. $r_{arrival}$ is the reward for UAVs that have reached the target positions. Furthermore, $\alpha_{goal}$ is the reward weight.

(3) $$r_{c}=\left\{\begin{array}{c} r_{collision}\;\;\;\;\;\;\;\;\;\;if\;crash\\ \alpha_{avoid}\cdot max\left(d_{safe}-d_{min},0\right)\;otherwise \end{array}\right.$$

where $r_{collision}$ is the collision penalty. $\alpha_{avoid}$ is the penalty weight. The $d_{safe}$ is the safe distance of UAVs. The $d_{min}$ is the minimum distance in a depth image.

#### 3.2.5. Structural Composition of UAV Control in AirSim

**Figure 2:**
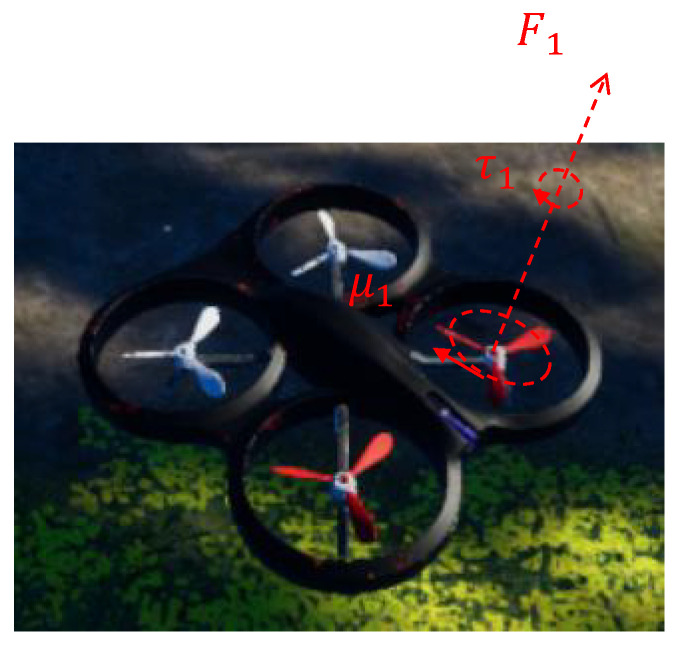
Quadrotor dynamic model visualization illustrating four rotors generating forces ($F_{1}$) and torques ($\tau_{1}$) under control inputs $u_{1}$.

The experimental platform utilizes AirSim’s component-based architecture for quadrotor control, featuring decoupled subsystems that enable efficient motion planning [29]. As shown in Figure 2, the policy network π(s) produces three-dimensional motion commands $a=[v_{x}^{cmd},v_{z}^{cmd},\omega^{cmd}]$, with each component being physically realized through rotor-generated aerodynamic forces.

The underlying physical model adheres to fundamental quadrotor dynamics described by:

(4) $$\left\{\begin{array}{c} m\cdot \frac{dv}{dt}=F-m\cdot g\\ I\cdot \frac{d\omega }{dt}=\tau \end{array}\right.$$

where *m* denotes UAV mass, v denotes the velocity vector, F denotes the total external force, g denotes the gravitational acceleration, *I* denotes the inertia matrix, ω denotes the angular velocity vector, and τ denotes the total external torque.

AirSim’s native PID regulation module handles flight control execution, with parameter settings detailed in Table 2 and UAV specifications in Table 3. This architecture separates high-level decision making from low-level actuation: the policy network focuses on optimal command generation while AirSim’s physics engine manages dynamic responses through PID-controlled motor outputs.

### 3.3. Architectural Overview

**Figure 3:**
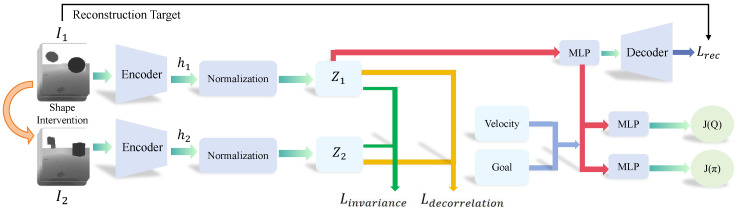
The illustration of our proposed method. To extract invariant causal representation, we intervene the shape of obstacles and maximize the mutual information through the $L_{invariance}$. In addition, we introduce the $L_{decorrelation}$ to ensure that different dimensions capture different information.

Our approach builds upon the previous work [13]. As shown in Figure 3, the framework comprises two parts: representation learning and policy learning. We construct a causal-representation-learning-structure-based autoencoder. It strives to learn representations that effectively reduce the information of obstacle shape. Subsequently, based on those causal representations along with the current speed and target position are inputted into policy network for strategy learning. Based on the current observation, the SAC algorithm will generate a series of actions for UAV control, which includes three-dimensional velocity adjustments. We adopted a learning paradigm of centralized training and distributed execution. Under this paradigm, the training phase has a global perspective, and the experience data collected by all drones during their interaction with the environment are aggregated into a central experience pool. This centralized learning enables the strategy to learn from the experience of all drones, which helps in the development of more effective and coordinated collision avoidance behaviors. In the execution phase, it is manifested as distributed operation, where each drone relies only on its own local observation information and independently uses this centrally trained and optimized shared policy network to make decisions and execute actions in real time, without the need for complex real-time communication or global state information between drones. This distributed execution method enhances the scalability and robustness of the system to single-point failures, allowing the trained strategy to be efficiently deployed in actual multi-drone operation scenarios.

In our formulation, we consider a set of N UAVs operating in a shared environment. At each timestep *t*, the *i*-th UAV receives an observation $o_{i}^{t}$ and computes an action $a_{i}^{t}$ accordingly, with the objective of moving from its current position toward its goal position $g_{i}^{t}$. Throughout the navigation process, each UAV has access only to its own observation and does not have access to the states of other UAVs. The observation vector for each UAV consists of three components, $o^{t}=\left[o_{i}^{t},o_{v}^{t},o_{g}^{t}\right]$, where $o_{i}^{t}$ represents images of the surrounding environment captured by the onboard vision sensor, $o_{v}^{t}$ denotes the current velocity of the UAV, and $o_{g}^{t}$ indicates the relative position of the goal. Given this partial observation $o^{t}$, each UAV independently and simultaneously samples an action $a^{t}$ which sampled from the policy π shared by all UAVs:

(5) $$a^{t}∼\pi_{\theta }\left(\cdot |o^{t}\right)$$

where θ is the parameter vector of the policy. The action $a^{t}$ corresponds to a velocity command that guides the UAV toward its goal while avoiding collisions, and remains in effect over the time horizon Δt until the next observation $o^{t}$ is received.

In the policy improvement step, we update the actor network by maximizing the loss function J(π), defined as follows:

(6) $$J\left(\pi \right)=E_{o∼B}\left[D_{KL}\left(\pi \left(a|o\right)∥Q\left(o,a\right)\right)\right]$$

where the term π(a|o) denotes the probability of taking action *a* given the observation *o*. The term Q(o,a) represents the target action distribution under the observation *o*.

We update the critic network by minimizing the loss function J(Q), formulated as follows:

(7) $$J\left(Q\right)=E_{(o,a,r,o^{\prime })∼B}\left[{\left(Q\left(o,a\right)-r-\gamma \bar{V}\left(o^{\prime }\right)\right)}^{2}\right]$$

where γ is the discount factor, $\bar{V}\left(o^{\prime }\right)$ is the target value function, and *r* denotes the obtained reward.

We adopt the loss function L(rec) to reconstruction image through updating the encoder $p_{\phi }$ and decoder ($z=g_{x}$):

(8) $$L\left(rec\right)=E_{x}\left[\log p_{\phi }\left(x|z\right)+\lambda_{z}{∥z∥}^{2}+\lambda_{\phi }{∥\phi ∥}^{2}\right]$$

where *x* is the depth image extracted from observations. $\lambda_{z}{∥z∥}^{2}$ and $\lambda_{\phi }{∥\phi ∥}^{2}$ are used to apply regularization constraints to *z* and model parameters ϕ. Both $\lambda_{z}$ and $\lambda_{\phi }$ are hyperparameters.

### 3.4. Extract Invariant Causal Representation

This section focuses on the learning of causal representations from images, which makes the optimal policy based on these representations to remain robust across various training domains. Effectively, this method aims to uncover and utilize the factors that contribute to successful actions. Some works [30] have demonstrated that we need to discover invariant mechanisms from multiple source domain data and identify hidden causal variables. Therefore, in this work, we conduct causal intervention on the shape of obstacles to construct multiple source domain data with different obstacles. Specifically, we pause the state of the UAV at the same instant and then change the shape of the obstacle by controlling Airsim. Through this method, we can obtain depth images of obstacles with different shapes at the same position from the perspective of the UAV.

We propose a causal representation learning (CRL) method based on an autoencoder (AE) to identify causal representations, as depicted in Figure 3. Using a depth camera mounted facing forward on the UAV, we sample two distinct sets of observations from the environment: $I_{1}$, containing depth images of the UAVs and original obstacles, and $I_{2}$, containing depth images of the UAVs and obstacles with modified shapes. These depth images capture the distance of object surfaces from the camera’s viewpoint. To incorporate temporal information, we stack four consecutive depth frames for each observation sequence. These stacked frames are then input into the encoder to obtain latent representations $h_{1}$ and $h_{2}$, corresponding to $I_{1}$ and $I_{2}$, respectively. Subsequently, we apply instance-dimensional normalization to $h_{1}$ and $h_{2}$ to ensure each feature dimension has a mean of 0 and a standard deviation of $\frac{1}{\sqrt{d}}$ (where *d* is the feature dimensionality). The normalization is performed using the following formula:

(9) $$z=\frac{h_{i}-\mu \left(h_{i}\right)}{\sigma \left(h_{i}\right)\times \sqrt{d}}$$

The obtained normalized $z_{1}$, $z_{2}$ are further used to maximize the mutual information through the invariance term:

(10) $$L_{invariance}=\left|\right|z_{1}-z_{2}{\left|\right|}^{2}$$

Intuitively, the invariance term is used to minimize the difference between two normalized representations.

Furthermore, previous work [31] has shown that multiple dimensions of representations share overlapping information. To ensure that different dimensions capture different information, we introduce the following decorrelation term:

(11) $$L_{decorrelation}=F\left(z_{1}z_{1}^{T},I\right)+F\left(z_{2}z_{2}^{T},I\right)$$

where $F\left(\cdot ,\cdot \right)={∥\cdot -\cdot ∥}_{F}^{2},{∥\cdot ∥}_{F}^{2}$ denotes the Frobenius norm. The symbol “·” denotes the input to the function *F* and *I* is an identity matrix. The decorrelation term helps prevent the trivial collapse where the same vector is produced for all inputs by trying to equate the off-diagonal elements of the auto-correlation matrix of each representation to 0.

The causal intervention process can be formalized using do-calculus:

(12) $$P\left(Y|do\left(X\right)\right)=\sum_{z}P\left(Y|X,Z=z\right)P\left(Z=z\right)$$

where do(X) denotes the intervention operation, *Z* represents the confounding variables.

The invariance loss can be derived from mutual information:

(13) $$I\left(Z_{1};Z_{2}\right)=E_{p(z_{1},z_{2})}\left[\log\frac{p(z_{1},z_{2})}{p\left(z_{1}\right)p\left(z_{2}\right)}\right]$$

Maximizing this mutual information ensures the learned representations are invariant to obstacle shapes.

The complete training procedure is formalized in Algorithm 1.
**Algorithm 1** Causal representation learning process.**Require:** Training environments $E_{1},\ldots ,E_{n}$
  1:  **for** each training iteration **do**
  2:  Sample batch $x_{i}$ from training experience // Gather diverse states before intervention
  3:  Apply shape intervention: $x_{i}^{intv}\leftarrow \mathrm{Intervention}\left(x_{i}\right)$ // Online causal intervention
  4:  Encode representations: $z_{i},z_{i}^{intv}\leftarrow f_{\theta }\left(x_{i}\right),f_{\theta }\left(x_{i}^{intv}\right)$
  5:  Compute invariance loss: $L_{invariance}=\left|\right|z_{i}-z_{i}^{intv}{\left|\right|}^{2}$ // Representation invariance enforcement
  6:  Compute decorrelation loss: $L_{decorrelation}=F\left(z_{1}z_{1}^{T},I\right)+F\left(z_{2}z_{2}^{T},I\right)$ // Dimensional decorrelation
  7:  Compute total representation loss: $L_{total}\leftarrow L_{invariance}+\lambda L_{decorrelation}$
  8:  Update encoder: $\theta \leftarrow \theta -\eta \nabla_{\theta }L_{inv}$
  9:  Train policy network with $\left\{z_{i}\right\}$
10:  **end for**


### 3.5. Causal Identifiability Analysis

**Figure 4:**
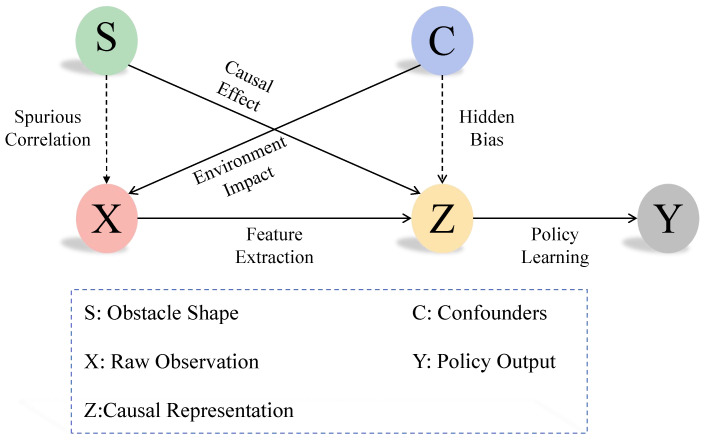
Structural causal model characterizing the collision avoidance system. Observed variables (*S*, *X*, *Y*) represent obstacle shapes, sensor inputs, and control commands, respectively. Latent variable *Z* denotes the causal representation learned through intervention do(S), while *C* captures environmental confounders. Directed edges encode causal mechanisms: solid lines show the intended policy learning path S→Z→Y; dashed lines indicate spurious correlations S→X→Z and confounding paths C→X→Z. The model satisfies identifiability via Z⊥C|do(S), enabling generalization across environments.

As depicted in Figure 4, our structural causal model formalizes the key relationships between obstacle characteristics, sensor observations, and control policies. The identifiability of causal representation *Z* is guaranteed through our intervention mechanism and contrastive learning framework. We formally prove the identifiability via the following three aspects:

**Theorem** **1** (Backdoor Path Blocking). *Under intervention do(S=s), the confounding path C→X→Z shown by dashed arrows in Figure 4 is blocked by cutting off the natural data-generating process of S. This satisfies the backdoor criterion:*

(14) P(Z|do(S))=∫P(Z|S,C)P(C)dC=P(Z|S)

The intervention severs the spurious correlation between *S* and *C* (visualized by the red dashed path in Figure 4), ensuring that the learned representation *Z* only captures the causal relationship S→Z (solid blue arrow). This is implemented through our obstacle shape randomization in AirSim’s rendering pipeline.

**Theorem** **2** (Conditional Independence). *The invariance loss $L_{\mathrm{invariance}}$ enforces the independence structure shown in Figure 4’s caption:*

(15) Z⊥⊥C|do(S)⇒I(Z;C|do(S))=0

*where I(·) denotes mutual information. This conditional independence is achieved through the following:*

- *Instance-wise normalization (Equation (9)), removing environment-specific statistics.*
- *Intervention-invariant representation learning (Equation (10)), enforcing $∥z_{1}-z_{2}{∥}^{2}$ minimization across environments under the same intervention.*

The decorrelation loss $L_{\mathrm{decorrelation}}$ further ensures disentangled representations by enforcing orthogonality constraints on the latent space, corresponding to the absence of bidirected edges between *Z* nodes in Figure 4:

(16) $$E\left[z_{i}^{T}z_{j}\right]=\delta_{ij},\;\forall i\neq j$$

This eliminates redundant information channels that might encode confounding factors.

**Theorem** **3** (Identifiable Mechanism). *The structural equations satisfy the causal Markov condition shown in Figure 4:*

(17) $$Z=f\left(S,\varepsilon_{z}\right),\;\varepsilon_{z}⊥\left(S,C\right)$$

*where f is an invertible nonlinear function learned by the encoder. The additive noise model ensures the identifiability up to component-wise transformations, corresponding to the solid causal pathway S→Z→Y in our SCM.*

The combination of intervention-based data generation and constrained representation learning establishes a minimal sufficient statistic *Z* for policy decisions. As shown in the causal graph (Figure 4), this enables generalization through the invariant causal mechanism P(Y|do(Z)) rather than spurious correlations P(Y|X) mediated by the dashed paths.

## 4. Results

### 4.1. Simulation Environment and Experimental Setup

AirSim is a high-fidelity simulation platform widely used for UAV (unmanned aerial vehicle) testing. Given its robust capabilities, we have chosen AirSim as the foundation for our simulation framework to investigate UAV collision avoidance algorithms. The simulation experiments were conducted on a computer equipped with the Ubuntu 20.04 operating system and powered by a single NVIDIA RTX 4090 GPU. The hyperparameters employed in this study are thoroughly outlined in Table 4.

### 4.2. Evaluation Metrics and Experimental Scenarios

To evaluate the efficacy of the proposed methodology, the following performance indicators were established:

- Success Rate: This metric quantifies the proportion of UAVs that successfully reach their destinations within a predefined time interval, devoid of any collisions.
- Excess Distance: This denotes the supplementary distance traversed by UAVs beyond the direct linear distance between the origin and the target destination.
- Success-weighted Path Length (SPL): This metric amalgamates the success rate with the efficiency of the navigated path, offering a holistic assessment of task accomplishment and path efficacy.
- Average Velocity: This is computed as the quotient of the aggregate flight path length and the time elapsed for the UAV.

The performance metrics are reported as mean/standard deviation (mean/std), calculated over multiple simulation trials for each experimental condition. The mean represents the average performance achieved across these trials, providing a measure of central tendency. The standard deviation (std) quantifies the variability or spread of the results.

**Figure 5:**
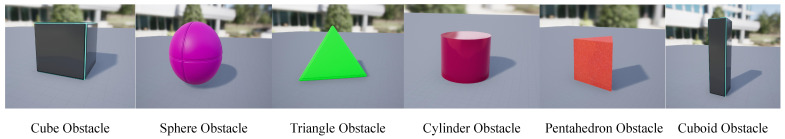
Simulation scenarios for evaluating the generalization ability. We set up six different shapes of obstacles in the playground scene during the testing phase.

As illustrated in Figure 5, to further evaluate the proposed method’s generalization capabilities, the CRL was tested against diverse obstacle shapes kept at a comparable size. The evaluated geometries encompassed simpler forms like cubes (side length—0.1 m) and spheres (diameter—0.1 m), alongside more complex structures including triangles, cylinders, pentahedrons, and cuboids (representative dimensions, 0.1 m × 0.1 m × 0.2 m). We randomly generate the initial and target positions of the Uavs and the specified positions of the obstacles in an area with a constant spatial dimension of 16 m×16 m×4 m configured in the simulation environment, containing eight UAVs and four stationary obstacles. In the training scenario, we choose the cube obstacle and the sphere obstacle as the seen obstacles of the UAV, while the others are the unknown obstacles during testing. In the training process, we fix the position of the obstacles in each frame; then, after the image data of the cube obstacle are obtained by the UAV, the original positions of the UAV and the obstacle are kept, and the shape of the obstacle is changed to that of the sphere obstacle, so as to achieve the purpose of shape intervention.

### 4.3. Performance Comparison

#### 4.3.1. Evaluation Obstacle Shapes

We compared our method against the SAC + RAE baseline across various obstacle scenarios under clear conditions to rigorously evaluate its generalization capabilities when faced with diverse object geometries. As quantitatively detailed in Table 5, our causal representation learning (CRL) approach significantly outperforms the baseline. This superiority is particularly evident when encountering previously unseen obstacle shapes such as triangles, cylinders, pentahedrons, and cuboids. For instance, in scenarios involving cuboid obstacles, CRL achieved a notable 6.2% higher success rate and a 3.7% improvement in SPL. These results highlight the effectiveness of learning true causal representations. By intervening on obstacle shape during training and enforcing invariance in the learned feature space, CRL learns features that correspond to the fundamental presence and properties of obstacles rather than overfitting to superficial characteristics like specific shapes (e.g., cubes or spheres) encountered during training, while the baseline method, SAC + RAE, struggles with novel geometries due to its reliance on potentially spurious correlations learned from the training distribution, CRL maintains robust and adaptive performance.

Furthermore, we challenged the models with randomly generated scenes where four different unseen obstacles were simultaneously present. The results from this “Mixed” scenario, also presented in Table 5, demonstrate CRL’s strong generalization capabilities, achieving a 7.1% higher success rate than the baseline. This underscores the ability of our method to handle complex environments with a variety of concurrently encountered novel objects, suggesting that the learned causal features provide a versatile foundation for decision making.

A nuanced observation pertains to the “Extra Distance” metric. For some unseen obstacles, such as cuboids, CRL resulted in a slightly longer flight path (1.851 m compared to 1.635 m for SAC + RAE). This suggests that the agent, guided by its understanding of core causal features, may adopt slightly more cautious or deliberative paths when navigating unfamiliar obstacles. This behavior prioritizes successful avoidance and mission completion over the shortest possible path, a strategy that might be favored by an overfitted baseline potentially underestimating risks associated with novel geometries. Nevertheless, the consistently higher average velocity observed for CRL across most scenarios indicates that, once decisions are made based on these robust causal features, the agent executes its actions efficiently. The ability to maintain good operational speed while navigating cautiously around novel objects points to a well-balanced policy learned by CRL. This careful yet efficient navigation is crucial for practical UAV applications where both safety and mission timeliness are paramount.

#### 4.3.2. Evaluation Under Variable Weather Conditions

**Figure 6:**
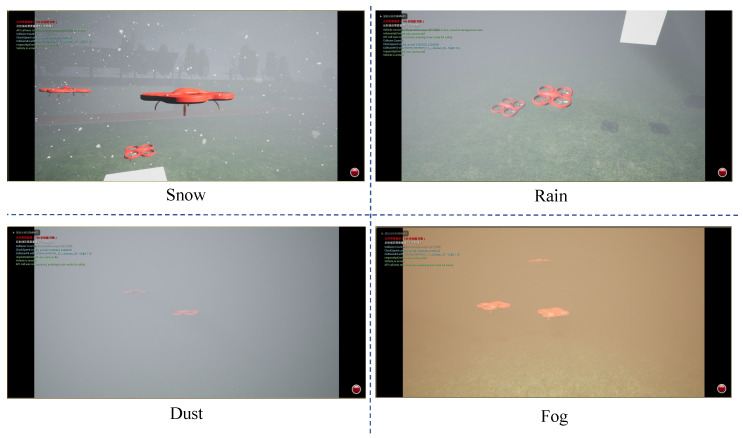
Simulation scenarios under variable weather conditions. The figure displays four different weather scenarios: snow, rain, dust, and fog, used to evaluate the generalization ability of our proposed method.

We conducted comprehensive testing of our method under diverse weather conditions while maintaining fixed cube-shaped obstacles, as shown in Figure 6.

The results in Table 6 underscore CRL’s robustness in challenging weather conditions. Compared to SAC + RAE, CRL shows marked improvements in success rate and SPL across snow, dust, and fog scenarios (e.g., an 11.6% increase in success rate in fog). This suggests that the causal representation learning helps the agent disentangle relevant obstacle information from sensor noise and visibility variations introduced by weather; while SAC + RAE’s performance degrades significantly as visual inputs become corrupted, CRL’s focus on invariant features provides a more stable basis for navigation policy. The consistently longer ’Extra Distance’ reinforces the idea that CRL prompts more path adjustments, likely to compensate for the increased uncertainty in perception under adverse conditions, but crucially, these adjustments lead to a much higher overall task success rate.

The results show our method maintains superior average velocity when navigating through static cube obstacles under changing weather, which is critical for mission efficiency. However, similar to previous evaluations, our method exhibits longer extra distances. This suggests UAVs require more frequent path adjustments to compensate for weather-induced sensor noise and visibility changes, ultimately increasing total travel distance.

#### 4.3.3. More Evaluation Baselines

To thoroughly assess the generalization capability of the CRL approach, we conducted a comparative analysis against several established deep reinforcement learning (DRL) generalization techniques. Specifically, CRL was benchmarked against three prominent baselines: the augmentation-based AutoAugment [32], the regularization-based DrAC [33], and the attention-based SE [34]. All methods were evaluated in a simulated playground environment containing four previously unseen cuboid obstacles. As shown in Table 7, CRL demonstrates clear advantages over the baselines. It achieves the highest success rate and SPL, outperforming AutoAugment, DrAC, and SE. Furthermore, CRL achieves this high success rate with notable efficiency, while its extra distance is comparable to the other baselines, its average velocity (0.860 m/s) is substantially higher than that achieved by AutoAugment (0.398 m/s), DrAC (0.379 m/s), and SE (0.377 m/s). This suggests that by focusing on invariant causal features, CRL not only improves the reliability of collision avoidance but also enables more decisive and efficient navigation when faced with unfamiliar scenarios. In summary, when evaluated against leading generalization strategies on previously unseen obstacles, the proposed CRL method achieved superior performance—delivering the highest success rate and SPL while maintaining competitive efficiency—validating its effectiveness in enhancing generalization for DRL-based collision avoidance.

#### 4.3.4. Ablation Experiment

An ablation study was performed to assess the individual contributions of the invariance loss ($L_{invariance}$) and the decorrelation loss ($L_{decorrelation}$) to the model’s performance, with results summarized in Table 8. The baseline model, without either of these loss components, achieved a success rate of 68.2%. Introducing only the decorrelation loss ($L_{decorrelation}$) resulted in a modest improvement, increasing the success rate to 69.6%. When only the invariance loss ($L_{invariance}$) was utilized, a more significant increase in performance was observed, with the success rate reaching 70.3%. However, the most substantial improvement was achieved when both the invariance and decorrelation losses were applied concurrently, yielding the highest success rate of 74.4%. In addition, under the condition of only $L_{invariance}$ or $L_{decorrelation}$, the drone needs to fly more distance and slower speed. This shows that, although $L_{invariance}$ and $L_{decorrelation}$ can each bring performance gains to the multi-drone collision avoidance task, the organic combination of the two is crucial to maximizing the ability of the agent to learn effective causal representations and ultimately achieve excellent generalization performance, mission success rate, and navigation efficiency.

#### 4.3.5. Scalability Analysis

**Figure 7:**
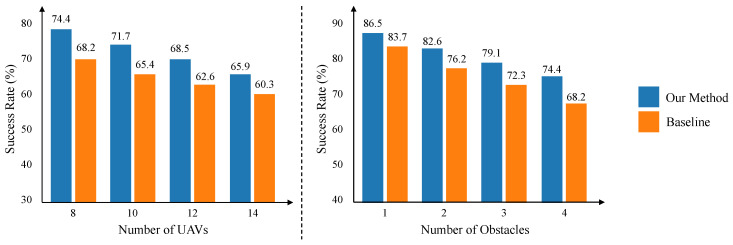
Scalability analysis experiments. Performance comparison between our proposed CRL and the baseline model under conditions with different numbers of UAVs and obstacles. The success rate is used as the evaluation metric.

To evaluate the scalability of proposed approach, we set up scenes with different numbers of UAVs and obstacles independently within a playground scene that includes four unseen cuboid obstacles. As shown in Figure 7, our method consistently exceeds the performance of SAC + RAE, demonstrating its superior robustness and scalability.

### 4.4. Visualization of Trajectory

**Figure 8:**
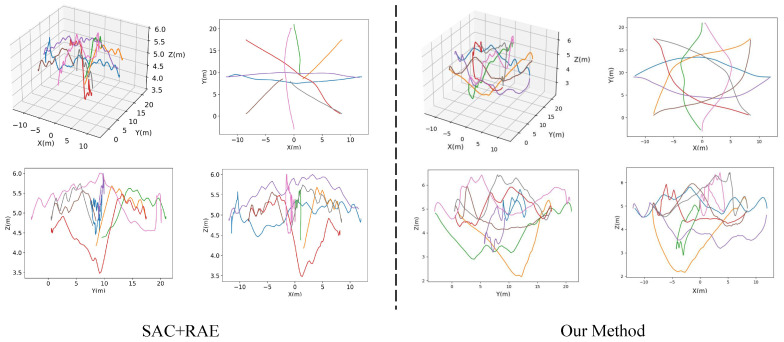
Visualization of UAVs trajectories in both perspective and three-view illustrations. We employ various colors to depict the trajectories of distinct UAVs.

To visually illustrate the collision evasion efficacy of our methodology, graphical representations of UAV flight paths are presented through perspective and triaxial orthographic projections. As exhibited in Figure 8, the proposed framework manifests enhanced trajectory optimization proficiency and obstacle navigation performance when contrasted with SAC + RAE architecture. This comparative analysis substantiates the operational superiority of our algorithm in multi-agent coordination scenarios.

**Figure 9:**
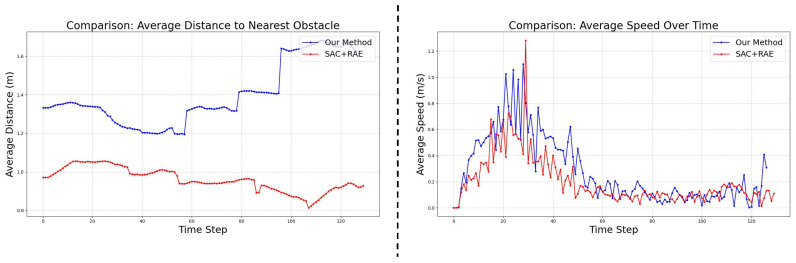
One round average extra distance contrast as well as average speed contrast for baseline and our method.

Figure 8 illustrates the scalability of our approach. As the number of UAVs or obstacles increases, the performance gap between CRL and the baseline widens. For instance, with 14 UAVs, CRL maintains a 65.9% success rate compared to the baseline’s 60.3%. This superior scalability indicates that CRL’s learned invariant representations are more robust to the increased complexity and potential for spurious correlations inherent in denser multi-agent scenarios. Focusing on fundamental causal factors allows the policy to remain effective even when interactions become more frequent and intricate. In addition, we compare the average extra distance in one round as well as the average speed of baseline and our method. The results are shown in Figure 9, where our method has advantages in average extra distance and average speed.

## 5. Conclusions

This paper presents a novel causal representation learning (CRL) method to enhance the generalization ability of deep reinforcement learning (DRL) models for multi-UAV collision avoidance. By intervening in the shape of obstacles and maximizing mutual information through the invariance term, our approach effectively identifies invariant causal representations from images. The introduction of the decorrelation term ensures that different dimensions of the representation capture distinct information, further improving the robustness of the learned policy. Extensive experiments demonstrate that our CRL method significantly outperforms state-of-the-art methods in various testing scenarios, including different obstacle shapes and variable weather conditions. The results highlight higher success rates, improved SPL (success weighted by path length), and faster average velocities; while our method shows promise, future work will focus on addressing current limitations, such as enhancing adaptability to background changes and validating the learned strategies in real-world settings. Additionally, we plan to explore more powerful disentanglement methods and advanced causal intervention strategies to further improve the generalization capabilities of this approach.

## 6. Discussion

The principal aim of our methodological framework was to assess the CRL technique’s capacity to mitigate the generalization challenge inherent in DRL frameworks when encountering unforeseen obstacles. Empirical findings substantiate the efficacy and preeminence of the CRL methodology in optimizing generalization capabilities across diverse evaluative contexts, confirming its operational superiority in cross-environmental adaptation tasks. However, several limitations in our current work warrant further investigation. Firstly, our model’s adaptability to background changes is limited, and future work should focus on improving this aspect. Secondly, our training and evaluation were conducted exclusively in simulated environments due to hardware constraints; while simulated environments provide a controlled setting for initial testing, real-world validation is crucial for assessing the practical applicability and robustness of the learned strategies. Future work should aim to deploy and evaluate our CRL method in real-world settings to bridge the gap between simulation and real-world deployment. In addition to addressing these limitations, future work will explore more powerful disentanglement methods and advanced causal intervention strategies to further enhance the generalization capability of our CRL approach. We also plan to apply the CRL method to other DRL learning tasks and investigate its potential in various application domains.

## Figures and Tables

**Table 1:** Definitions of some key notations.

Notations	Definitions
*S*	State space in POMDP
*A*	Action space in POMDP
$P(s^{\prime }|s,a)$	Transition probability function in POMDP
R(s,a)	Reward function in POMDP
Ω	Observation space in POMDP
O(o|s)	Observation function in POMDP
O	UAV’s observation space, O=[I,V,G]
*I*	Accumulation of four consecutive depth images (part of O)
*V*	Current velocity of the UAV (part of O)
*G*	Euclidean distance to the target destination (part of O)
*a*	Action vector, $a=[v_{x}^{\mathrm{cmd}},v_{z}^{\mathrm{cmd}},v_{\omega }^{\mathrm{cmd}}]$
$v_{x}^{\mathrm{cmd}}$	Commanded forward velocity
$v_{z}^{\mathrm{cmd}}$	Commanded lateral velocity
$v_{\omega }^{\mathrm{cmd}}$	Commanded angular velocity
$I_{1}$	Depth images of UAVs and original obstacles
$I_{2}$	Depth images of UAVs and obstacles with modified shapes (after intervention)
$z_{1},z_{2}$	Normalized latent representations from $I_{1},I_{2}$, respectively,
$L_{\mathrm{invariance}}$	Invariance loss, $\left|\right|z_{1}-z_{2}{\left|\right|}^{2}$
$L_{\mathrm{decorrelation}}$	Decorrelation loss to ensure different dimensions capture different information
$L_{\mathrm{rec}}$	Reconstruction loss for the autoencoder

**Table 2:** PID parameter configurations for flight control.

Controller Type	$K_{P}$	$K_{I}$	$K_{D}$
Linear-velocity control	0.2	2.0	0.0
Angular-velocity control	0.25	0.0	0.0

**Table 3:** Characteristics of the quadrotor platform.

Item	Parameter
Weight	0.8 kg
Number of rotors	4
Minimum motor thrust	0
Maximum motor thrust	1
Minimum tilt throttle	0.05
Maximum angular velocity	2.5 rad/s
Dimensions (m)	0.2413 × 0.1143 × 0.0762

**Table 4:** Hyperparameters for policy training.

Parameter Name	Value
Batch size	128
Max episodes	150
Update times	400
Replay buffer B capacity	20,000
Discount γ	0.99
Learning Rate	$10^{-3}$
Critic’s target update frequency	2
Critic’s Q-function soft-update rate $\tau_{Q}$	0.01
Critic’s encoder soft-update rate $\tau_{enc}$	0.05
Actor’s update frequency	2
Actor’s log stddev bounds	[−10, 2]
Optimizer	Adam

**Table 5:** Performance (as mean/std) comparison with different obstacle shapes.

Obstacle Shape	Unseen/Seen	Method	Success Rate (%)	SPL (%)	Extra Distance (m)	Average Velocity (m/s)
Cube	Seen	SAC + RAE Our method	67.6 74.4 (↑ 6.8)	58.3 62.5 (↑ 4.2)	1.483/1.436 1.542/1.519	0.771/0.158 0.806/0.125
Sphere	Seen	SAC + RAE Our method	68.3 73.4 (↑ 5.1)	58.4 62.2 (↑ 3.8)	1.680/1.709 1.735/1.718	0.783/0.160 0.814/0.132
Triangle	Unseen	SAC + RAE Our method	72.5 76.3 (↑ 3.8)	62.1 64.4 (↑ 2.3)	1.535/1.622 1.815/1.802	0.775/0.164 0.809/0.135
Cylinder	Unseen	SAC + RAE Our method	67.6 72.1 (↑ 4.5)	58.6 61.7 (↑ 3.1)	1.646/1.621 1.853/1.810	0.783/0.155 0.810/0.138
Pentahedron	Unseen	SAC + RAE Our method	67.3 73.8 (↑ 6.5)	56.7 61.8 (↑ 5.1)	1.575/1.546 1.651/1.623	0.784/0.153 0.813/0.130
Cuboid	Unseen	SAC + RAE Our method	68.2 74.4 (↑ 6.2)	59.3 63.0 (↑ 3.7)	1.635/1.578 1.851/1.820	0.799/0.131 0.803/0.136
Mixed	Unseen	SAC + RAE Our method	67.1 74.2 (↑ 7.1)	60.4 62.9 (↑ 2.5)	1.646/1.601 1.858/1.826	0.801/0.129 0.805/0.131

**Table 6:** Comparison of the performance (as mean/std) of cube obstacles under different weather conditions.

Weather Type	Unseen/Seen	Method	Success Rate (%)	SPL (%)	Extra Distance (m)	Average Velocity (m/s)
Rain	Seen	SAC + RAE Our method	63.6 69.5 (↑ 5.9)	51.2 54.3 (↑ 3.1)	2.653/3.143 2.163/3.785	0.424/0.137 0.374/0.115
Snow	Unseen	SAC + RAE Our method	61.8 71.2 (↑ 9.4)	51.1 56.4 (↑ 5.3)	**2.699/2.685** 2.432/3.773	0.406/0.131 0.385/0.112
Dust	Unseen	SAC + RAE Our method	59.8 8.1 (↑ 8.3)	51.7 58.1 (↑ 6.4)	3.124/2.620 2.399/4.079	0.371/0.131 0.381/0.109
Fog	Unseen	SAC + RAE Our method	57.9 69.5 (↑ 11.6)	49.8 59.7 (↑ 9.9)	2.965/2.545 2.447/4.786	0.387/0.134 0.379/0.124

**Table 7:** Performance comparison of various methods in the testing scenario with four cuboid obstacles.

Method	Success Rate (%)	SPL (%)	Extra Distance (m)	Average Velocity (m/s)
SAC + RAE	68.2	59.3	1.635/1.578	0.799/0.131
+ AutoAugment [32]	68.8	61.2	1.505/2.881	0.398/0.131
+ DrAC [33]	69.8	61.5	1.538/2.693	0.379/0.122
+ SE [34]	70.1	61.6	1.564/2.648	0.377/0.122
+ Our method	74.4	62.5	1.542/1.519	0.860/0.125

**Table 8:** Ablation study on different loss functions.

$L_{invariance}$	$L_{decorrelation}$	Success Rate(%)	SPL(%)	Extra Distance (m)	Average Velocity (m/s)
		68.2	59.3	1.635/1.578	0.799/0.131
✓		69.6	60.1	1.584/1.539	0.814/0.129
	✓	70.3	60.4	1.603/1.554	0.821/0.122
✓	✓	74.4	62.5	1.542/1.519	0.860/0.125

## Data Availability

The data presented in this study are available upon request from the corresponding authors.

## Abbreviations

The following abbreviations are used in this manuscript:

UAV	unmanned aerial vehicle
SLAM	simultaneous localization and mapping
DRL	deep reinforcement learning
RAE	regularized autoencoder
CRL	causal representation learning
